# The Predictor Role of Perceived Social Support and Spiritual Intelligence in Hope Among Thalassemia Patients

**DOI:** 10.21315/mjms2020.27.3.8

**Published:** 2020-06-30

**Authors:** Nilofar Pasyar, Masoume Rambod, Zahra Behrouzi

**Affiliations:** 1 Community Based Psychiatric Care Research Center, Shiraz University of Medical Sciences, Shiraz, Iran; 2 School of Nursing and Midwifery, Shiraz University of Medical Sciences, Shiraz, Iran; 3 Student Research Committee, Shiraz University of Medical Sciences, Shiraz, Iran

**Keywords:** hope, thalassemia, perceived social support, spiritual intelligence

## Abstract

**Background:**

Thalassemia is a prevalent chronic blood disorder, which is accompanied with a wide range of challenges. This study aimed to determine the predictor role of perceived social support and spiritual intelligence in hope among thalassemia patients.

**Methods:**

This cross-sectional study was conducted on 300 thalassemia patients. The data were collected using Miller hope scale, spiritual intelligence scale, and personal resource questionnaire. The data were analysed using Pearson’s correlation coefficient and multiple regression analysis.

**Results:**

The results indicated that the mean score of hope was 173.38 (standard deviation [SD] = 24.6). Besides, 86.7% of the patients showed high levels of hope. The mean score of spiritual intelligence was 134.66 (SD = 18.07). In addition, 91.7% of the patients showed moderate levels of spiritual intelligence. The mean score of social support was 127.87 (SD = 20.19) and the patients showed high social support levels. The results revealed a significant association between hope and perceived social support (*r* = 0.65, *P* < 0.001) as well as between hope and spiritual intelligence (*r* = 0.63, *P* < 0.001). Moreover, perceived social support (*b* = 0.43, *P* < 0.001) and spiritual intelligence (*b* = 0.37, *P* < 0.001) were the predictors of hope among thalassemia patients.

**Conclusion:**

Considering the study results, in addition to common treatments for thalassemia, policymakers’ are recommended to pay attention to spiritual intelligence and more importantly social support to enhance thalassemia patients’ hope.

## Introduction

Nowadays, incidence of chronic diseases in all age groups, socioeconomic classes and cultures has been considered to be a major health problem in developed countries. The World Health Organization (WHO) has introduced thalassemia as the most prevalent chronic genetic disorder in 62 countries around the globe (1). According to WHO’s reports, nearly 60,000 infants with major thalassemia are born annually, which has been regarded as a global problem (2). The probability of birth of an infant with thalassemia has been estimated to be one per 300 deliveries in Iran. Moreover, almost 1,500 new patients are added to this measure annually. In this country, the largest populations of patients with major thalassemia belong to Mazandaran and Fars Provinces (3).

Similar to patients with other chronic and disabling diseases, thalassemia patients are engaged in various psychological, economic and social problems due to lifelong treatments. The outcomes of the disease include decreased quality of life, hopelessness, social isolation, familial tensions and social stigmatisation (4, 5), which increase the patients’ need for support on the part of others (6).

Social support refers to establishment of social interactions and empathic relations, which result in a safe network for patients, eventually helping them cope with conditions and problems and have a better feeling towards themselves (7). In addition to the positive effect of social support, patients’ perception in this area should be taken into consideration. In other words, patients’ perception and attitude towards the received support are more important than the amount of support provided for patients (8).

Intelligence provides people with the ability to cope with problems and solve them compatibly. Additionally, spirituality refers to belief in the supreme power, including seeking for the meaning of life, self-awareness and self-knowledge, as well as feeling of responsibility towards and unity with others. The combination of intelligence and spirituality has resulted in the creation of spiritual intelligence construct (9). Spiritual intelligence enables individuals to cope with difficulties, pains and failures and provides them with higher compatibility (10, 11). People with higher spiritual intelligence possess higher flexibility, self-awareness, and capacity for dealing with difficulties. Moreover, because of their holistic view, they seek for answers to basic questions of life. This can promote hope and well-being among patients (9).

Unpleasantness, length and repetition of treatment regimens for thalassemia patients can affect their other dimensions of life (12). Hope can play a critical role in increasing the patients’ ability to cope with their problems through the treatment process, accelerating the recovery process and enhancing the probability of recovery (13). Generally, hope has been considered to be a vital factor for dealing with stress and increasing the life quality during stressful life periods (13–15). Hope can definitely affect individuals’ thoughts, emotions, and achievements, as well. Despite the importance of hope among thalassemia patients, it has been less taken into account in theoretical and scientific investigations (11–15). Furthermore, social support has been considered to be the strongest coping strategy, which provides individuals with the ability to successfully cope with stressful conditions and tolerate problems (16). Moreover, regarding spiritual resources, spiritual intelligence refers to people’s capabilities, including compatibility and problem-solving behaviours (17).

A review of the literature revealed that limited studies have been conducted on hope among thalassemia (13), and hemodialysis patients (18) and students (14). Additionally, only a few studies have investigated perceived social support among patients with chronic diseases, such as chronic renal failure (6, 19). On the other hand, studies performed on thalassemia patients have focused on their health and quality of life (11, 12). Considering perceived social support, spiritual intelligence and hope among thalassemia patients, only one research has been carried out on perceived social support among these patients (20). Besides, a study conducted by Muazzam and Sumble in 2013 (21) assessed the perception of hope among thalassemia patients. Thus, limited studies have been performed on perceived social support, spiritual intelligence, and hope in thalassemia patients. Hence, the present study aims to determine the predictor role of perceived social support and spiritual intelligence in hope among thalassemia patients.

## Methods

This cross-sectional study was conducted on thalassemia patients referred to the outpatient thalassemia ward in Shahid Dastgheib Hospital in Shiraz, Fars Province, Iran from February 2019 to May 2019. Regarding the lack of similar studies in this field and considering *r* = 0.25, type one error = 0.05 and power = 80%, a 130-subject sample size was required for the study. However, considering *r*-values in age, education and sex subgroups, the sample size was needed to be doubled. Therefore, 300 thalassemia patients were selected via convenience sampling. The inclusion criteria of the study were definite diagnosis of thalassemia based on hemoglobin electrophoresis, having an active profile in the hospital, regular referral to the hospital during six months, dependence on transfusion, age above 18 years, ability to read, write and understand Persian, and willingness to cooperate in the research. The exclusion criteria were experiencing acute conditions and hospitalisation during the past month, suffering from known psychological disorders, not completing the study questionnaires and not being willing to continue participation in the study. The incomplete data were excluded from the study, as well. The study data were collected using a demographic information form, Miller hope scale (22), Weinert and Brandt’s personal resource questionnaire (23), and Amram and Dryer’s spiritual intelligence scale (24). The demographic information form included such variables as age, sex, marital status, education level, and occupation.

Miller hope scale was developed by Miller and Powers (22) and contained 48 items responded through a five-point Likert scale ranging from 1 = completely disagree to 5 = completely agree. Additionally, 14 items were scored reversely (22). Scores 48–96, 96–144, and > 144 represented low, moderate and high hope levels, respectively (9). It should be noted that the average score of the scale was 144. The reliability of the scale was reported to be 80% by Miller and Powers (22) and 0.95 by Gholami et al. (11). In the present study, the reliability of the scale was calculated as 0.885.

The spiritual intelligence scale was developed by Amram and Dryer (24, 25) and contained 39 items. The items were responded through a six-point Likert scale with the following options: never, rarely, relatively low, relatively high, very high and always. Accordingly, scores 39–78, 78–156 and > 156 indicated low, moderate and high spiritual intelligence levels, respectively (25, 26). The reliability of the scale was approved by Cronbach’s alpha coefficient of 0.97 in the study by Amram and Dryer (24) and 0.90 in the one by Khodadady et al. (27). In the present study, the reliability of the scale was confirmed by Cronbach’s alpha = 0.851.

Weinert and Brandt’s (23) personal resource questionnaire was developed in 1981 to assess perceived social support. The questionnaire included 25 items responded via a seven-point Likert scale ranging from 1 = completely dissatisfied to 7 = completely satisfied. It should be noted that negative questions were scored reversely. In this questionnaire, higher scores represented higher perception of support. Accordingly, scores 126–175, 76–125, and 25–75 indicated high, moderate and low social support, respectively (28). The validity of this questionnaire was confirmed by Rambod et al. (19, 28). Its face validity was approved, as well. Indeed, its test-retest reliability was found to be 0.85. In the present study, the reliability of the questionnaire was approved by Cronbach’s alpha = 0.875.

After all, the data were analysed using the SPSS software, version 22. Descriptive statistics included mean, SD, relative frequency and percentage. Pearson’s correlation coefficient was used to assess the correlations between the variables. Finally, the predictor role of social support and spiritual intelligence was evaluated using multiple regression analysis. *P* < 0.05 was considered to be statistically significant.

## Results

The mean age of male and female patients was 27.4 (SD = 5.56) and 30.42 (SD = 6.93) years, respectively. According to Table 1, 56.67% the participants were female. Most of the patients (75.25%) were single. Considering education level, five patients (1.70%) were illiterate, 17 (5.66%) had primary school degrees, 103 (34.32%) had middle school and high school degrees, and 175 (58.32%) had academic degrees. With regard to occupation, 51.33% of the participants were jobless (Table 1).

Kolmogorov-Smirnov test was used to determine the normal distribution of the data. The results showed that hope, spiritual intelligence, and perceived social support followed normal distribution.

The total mean score of hope was 173.38 (SD = 24.6). Moreover, 86.7%, 12.3% and 1% of the patients had high, moderate and low hope levels, respectively.

The mean score of spiritual intelligence was 134.66 (SD = 18.07). Among the patients, 91.7% had moderate and 8.3% had high spiritual intelligence levels.

The mean score of perceived social support was 127.87 (SD = 20.19). Considering the fact that the scores of social support ranged from 25 to 175 and higher scores represented higher perception of social support, the participants benefitted from a high social support level.

The results of Pearson’s correlation coefficient presented in Table 2 showed a significant, positive association between hope and perceived social support (*r* = 0.654, *P* < 0.001). A significant association was also observed between hope and spiritual intelligence (*r* = 0.630, *P* < 0.001). Therefore, as the patients’ perceived social support and spiritual intelligence increased, their hope increased, as well.

The results of regression analysis presented in Table 3 revealed the predictor role of spiritual intelligence (*P* < 0.001) and perceived social support (*P* < 0.001) in hope. The regression model for investigation of the linear relationship between hope, and spiritual intelligence and social support can be written as follows:

Model: Patients’ hope = 37.85 + 0.51 (spiritual intelligence) + 0.53 (perceived social support).

According to this model, a unit increase in the score of spiritual intelligence was accompanied with a 0.51 unit (95% CI: 0.37–0.64) increase in the score of hope (*P* < 0.001). Moreover, a unit increase in the score of perceived social support was accompanied with a 0.53 unit (95% CI: 0.4–0.65) increase in the score of hope (*P* < 0.001). In addition, the value of β was higher in the score of social support (0.43) than in the score of spiritual intelligence (0.37). Therefore, perceived social support was more effective in the patients’ hope compared to spiritual intelligence. Considering the value of *R*^2^ in the model (*R*^2^ = 0.514), the scores of perceived social support and spiritual intelligence explained nearly 51.4% of the changes in the patients’ hope scores. In other words, 51.4% of the changes in the score of hope were attributed to these two factors, and the rest of changes resulted from other unknown factors that were not entered into the model.

## Discussion

This study was conducted on 300 thalassemia patients referred to Shahid Dastgheib Hospital, Shiraz in 2019 in order to assess the predictor role of perceived social support and spiritual intelligence in hope. The results indicated that the mean score of hope was 173.38 (SD = 24.6) and 86.7% of the patients had high hope levels. In the same vein, Perveen (29) reported high hope levels among thalassemia patients in Pakistan. Dehbashi et al. (30) and Madani et al. (31) also assessed hope among the patients referred to hemodialysis centers and those who suffered from cancer and showed that the mean score of hope was 77.17 (ranging from 12 to 36), which represented a high hope level. In contrast, Hejazi et al. (32) revealed that 17.7% of the hemodialysis patients reported moderate hope levels. Orlandi et al. (33) also demonstrated a low hope level among hemodialysis patients. The difference among the results might be attributed to variations in the type of diseases and the provided services. Life expectancy refers to the probability of increased life span, such a way that individuals spend more years in a healthy and active condition. The quality of services provided for thalassemia patients and creation of electronic health records for patients with major thalassemia in the recent years have inclined individuals to build their future and have plans and be willing to achieve their goals and cope with obstacles (34). Hope is an internal force, which richens patients’ lives and creates optimism towards the disease, pain, and perspective of death (31). On the other hand, there may be synergetic mechanisms in various psychological stages of coping with thalassemia, justifying the high score of hope.

In the present study, the mean score of social support was 127.87 (SD = 20.19), indicating a high social support level. Evidence has indicated that family was the most and friends were the least important sources of social support for adult patients with major thalassemia (35). In line with the findings of the current research, studies conducted by Hooshmandi et al. in Bushehr (20), Jafari et al. among adolescents with chronic diseases such as thalassemia in Tehran (36) and Maheri et al. (35) among adults with thalassemia showed high social support levels among patients with thalassemia (20). Khurana et al. (37) also showed Indian adolescents’ financial and emotional dependence on their parents. Hence, it can be concluded that thalassemia patients believed that they were cared, loved, valued, and respected in a network of mutual relationships and commitments. Based on the social cohesion theory and the symbolic interactionist theory, social participation supports people against disruptive and turbulent functions and affects their psychological well-being by providing the ground for formation of stable identities and positive self-understanding (38).

In the current study, the mean score of spiritual intelligence was 134.66 (SD = 18.07). In addition, 91.7% of the patients showed moderate spiritual intelligence levels. On the contrary, Merati Fashi et al. (39) reported that spiritual intelligence was at moderate level in 32% and at high level in half of dialysis patients. According to King’s theory, the majority of thalassemia patients referred to Dastgheib Hospital in Shiraz had a moderate compatible mental ability based on transcendental aspects. This might be attributed to insufficient training.

In line with the results of the present study, studies conducted by Alipour et al. (40) on patients with type II diabetes, Madani et al. (31) on Iranian patients with cancer and Taei et al. (14) on patients with breast cancer revealed a positive correlation between social support and hopefulness. Pehlivan et al. (41) also performed a study on Turkish cancer patients and showed a negative relationship between hopelessness and perceived familial social support. According to the symbolic interactionist theory, social support is a coping strategy through which network members support each other while confronting with a problem. In this context, they believe that they are valued and cared by others (including friends and colleagues) and will be assisted in case of problems and discomfort. Such a support creates the necessary motivation in people and enables them to create opportunities to achieve desirable goals in spite of the existing barriers. In other words, it enhances their hope (38, 42).

The results of the current study demonstrated that increase in spiritual intelligence was accompanied with increase in life expectancy. Mohammadi et al. (43) also performed a research on dialysis patients in Tehran and indicated that spiritual intelligence training enhanced the patients’ purposefulness and orientation and promoted their personal growth conception, control over the environment, independence, and positive relations with others. In the same line, Dehbashi et al. showed a significant positive relationship between hope and spiritual well-being (30). Moreover, Vaezi (10) stated that spirituality improved individual, psychological, and social functions among female thalassemia patients. Spiritual intelligence is the ability, which strengthens people to try to make their dreams come true and promotes their daily function as well as physical and mental health (44). On the other hand, spiritual beliefs enable individuals to give meaning to difficulties, psychological pressures, and unavoidable losses in life and to build an optimistic viewpoint towards the next life that will be accompanied with tranquility (45).

The current study findings revealed that social support and spiritual intelligence scores explained nearly 51.4% of the changes in the patients’ hope scores. Up to now, no studies have assessed the simultaneous predictor roles of spiritual intelligence and perceived social support in hope. Nonetheless, Zeighmi et al. (46) reported that increase in spiritual well-being resulted in an increase in hope and social support among adolescents with thalassemia. The role of perceived social support in spiritual intelligence and hope has been evaluated, as well. The results of the studies conducted by Alipour et al. in patients with type II diabetes (40), Madani et al. in Iranian patients with cancer (31), Taei et al. (14) and Pehlivan et al. (41) in patients with breast cancer showed a positive correlation between social support and hopefulness. Dehbashi et al. (30) also revealed a significant positive relationship between hope and spiritual well-being. Perceived social support enables individuals to achieve their dreams by providing positive impacts, feeling of predictability, stability in life situations, and promotion of well-being (47). Such ability enhances the patients’ hope.

One of the limitations of this study was lack of motivation in some patients and their families to take part in the research. However, the researcher tried to increase their motivation to complete the questionnaires by simply explaining the study objectives and describing the impact of the results on the patients’ quality of life. Future studies are recommended to assess the effect of social support on hope among thalassemia patients and their peers. Further studies are also suggested to determine the effective factors in hope as well as the effectiveness of various interventions in increasing hope among thalassemia patients and their caregivers.

## Conclusion

The study results revealed a correlation between hope and perceived social support. Therefore, measures should be taken to provide thalassemia patients with strong social support networks alongside the common treatments. In addition, considering the relationship between hope and spiritual intelligence among the patients, spiritual intelligence should be regarded as the core of preventive, educational, and personal growth interventions, particularly in unbearable conditions. The study findings also showed that social support and spiritual intelligence were the predictors of hope among thalassemia patients. Indeed, social support was more effective in hope compared to spiritual intelligence. Hence, policymakers are recommended to pay special attention to both dimensions, with more emphasis on social support, so as to enhance thalassemia patients’ hope.

## Figures and Tables

**Table 1:** The demographic characteristics of the thalassemia patients in this study

Variables	*n* (%)^a^
Gender	
Male	130 (43.33)
Female	170 (56.67)
Marital status	
Single	222 (75.25)
Married	64 (21.70)
Widowed	4 (1.35)
Divorced	5 (1.70)
Education level	
Illiterate	5 (1.70)
Primary school	17 (5.66)
Middle and high schools	103 (34.32)
Academic	175 (58.32)
Occupation	
Jobless	154 (51.33)
Employee	36 (12.00)
Self-employed	87 (29.00)
Other	23 (7.67)

Note:

a Frequency (%)

**Table 2:** The association between hope, and perceived social support and spiritual intelligence

Variables		Hope
Hope	Pearson’s correlation	1
	*P*-value	
Perceived social support	Pearson’s correlation	0.654
	*P*-value	< 0.001
Spiritual intelligence	Pearson’s correlation	0.63
	*P*-value	< 0.001

**Table 3:** The results of linear regression analysis for investigating the predictor role of perceived social support and spiritual intelligence in the thalassemia patients’ hope

Model	Non-standard coefficients		Beta standard coefficients	*t*	*P*-value	95% CI^a^

	B	SE				
Model constant	37.85	7.814	-	4.84	0.001 >	-
Spiritual intelligence score	0.51	0.07	0.37	7.42	0.001>	0.37–0.64
Perceived social support score	0.53	0.06	0.43	8.56	0.001>	0.4–0.65

Note:

a Confidence interval
